# Virtual Reality and Augmented Reality Training in Disaster Medicine Courses for Students in Nursing: A Scoping Review of Adoptable Tools

**DOI:** 10.3390/bs13070616

**Published:** 2023-07-24

**Authors:** Camilla Elena Magi, Stefano Bambi, Paolo Iovino, Khadija El Aoufy, Carla Amato, Chiara Balestri, Laura Rasero, Yari Longobucco

**Affiliations:** 1 Department of Health Sciences, University of Florence, 50121 Firenze, Italy; camillaelena.magi@unifi.it (C.E.M.); stefano.bambi@unifi.it (S.B.); paolo.iovino@unifi.it (P.I.); carla.amato@unifi.it (C.A.); chiara.balestri@unifi.it (C.B.); l.rasero@unifi.it (L.R.); 2 Department of Experimental and Clinical Medicine, University of Florence, 50121 Firenze, Italy; khadija.elaoufy@unifi.it

**Keywords:** nursing students, paramedic students, training, virtual reality, augmented reality, disaster medicine, mass casualty incidents, competence, self-efficacy, learning immersion

## Abstract

Nurses and paramedics play a pivotal role when mass casualty incidents (MCI) occur, yet they often feel unprepared for such events. Implementation strategies for training activities, including virtual reality (VR) and augmented reality (AR) simulations, offer realistic and immersive learning experiences, enhancing skills and competencies for nursing students. The aim of this work was to investigate the adopted tools in studies on VR and AR simulations for training nursing and paramedic students in managing MCI. A scoping review was performed following the PRISMA-ScR statement, and the search strategy was conducted through five electronic databases from December 2022 to March 2023. Of 162 records identified, 27 full texts were screened and, six studies were included in this review. These studies involved students who were assigned to different training methods, including immersive VR simulation, written instruction, and traditional lecture. VR and AR and immersive simulation generally show promising evidence in enhancing practical skills and knowledge in MCI management. VR and AR showed to be promising in disaster education and preparedness training, offering different levels of immersiveness and engagement, encouraging active and experiential learning. Further research is needed to determine their long-term effectiveness. The choice of training method should consider program goals, target population, and available resources.

## 1. Introduction

In the last decades, disasters have recurred cyclically, putting human life and possessions at high risk [1]. Disasters can persist for hours or months and happen without much notice [1]. Pandemics, terrorist attacks, and natural disasters such as tsunamis, earthquakes, and storms are recent examples of multiple casualty incidents (MCI).

Nurses and paramedics play a vital role in disaster relief globally and much has been done since their profession’s inception [1,2]. These events are very stressful for frontline healthcare workers due to their workload increase, multitasking, and time pressures, as well as their need to deal with horrific situations and their own anxiety, uncertainty, and worries about how such calamities will affect their homes and families [3,4]. Thus, healthcare workers frequently experience extreme physical and psychological stress that exceeds what is typically supported by basic training and the healthcare system they work within [3].

Nevertheless, there is evidence that most nurses do not feel adequately prepared for a disaster in their community [1]. Disaster preparedness is defined as the capacity to adequately plan and react to a major catastrophe that strikes abruptly, with the goal of acting promptly to reduce damage and ensure the readiness of both personal and governmental countermeasures [5]. To obtain and maintain an adequate level of preparation, staff and nursing students should participate in the creation, evaluation, and simulation of disaster plans as training activities [1]. In addition, the training also shows benefits in willingness to participate in disaster response, defined as an individual’s voluntary inclination to participate in it [5,6]. In fact, even though nurses play a crucial role in disaster response, not all nurses are willing to take part in it, leading to a shortage of healthcare workers in disaster situations management. However, according to the social cognitive theory (SCT), which includes factors such as behavioral patterns and the person’s cognitive, affective, and physiological aspects, the willingness to participate can be improved with adequate training [7,8,9]. Nevertheless, SCT was widely applied in health professional education programs, showing the capacity to provide a faster and safer learning than the trial and error approach, encouraging collaborative learning, self-confidence, learning experiences and engagement [10].

For this reason, recent literature stressed the significance of disaster preparedness education and the necessity of disaster readiness at individual, family, and nursing curriculum levels for nursing students, together with the willingness to participate in disaster response [5]. In order to achieve this purpose, the best way to develop skills and competencies is through the design of educational training programs that could be ongoing, simple to access, interesting, and realistic, exposing the learner to high-fidelity simulations [1]. Furthermore, education for disaster preparedness should include the use of technology, such as virtual reality (VR) and augmented reality (AR), for their great potential to train healthcare professionals and students, in particular on self-efficacy, clinical reasoning capacity, learning immersion, and learning satisfaction [1,11,12].

From this perspective, traditional knowledge transmission is replaced by experiential learning, where knowledge construction occurs through active experimentation involving the analysis of a problem from multiple perspectives. This process is achieved by engaging in observation and reflection on the lived experience [13].

Regarding learning immersion, there is evidence that the higher the level of immersion that can be reached, the greater the effects on self-efficacy [14]. Moreover, these types of simulations are considered a financially advantageous and resource-conscious pedagogical option for nursing education [15]. For these reasons, in the last years, different typologies of VR and AR simulations were tested, adopting various tools [16].

VR is a technology that creates a simulated, three-dimensional environment that users can interact with using specialized equipment, such as headsets and controllers. It immerses users in a computer-generated environment that replicates real-world or imaginary scenarios [17]. AR, on the other hand, overlays digital information or virtual objects onto the real-world environment, enhancing the user’s perception of reality by adding computer-generated elements. AR is typically experienced through mobile devices, smart glasses, or heads-up displays [18].

According to literature, there are different VR and AR devices that provide different levels of immersion and consequently determine a different level of user involvement in the immersive experience.

Desktop virtual reality (DVRs) are devices such as video games or video simulations, in which the user interacts with a three-dimensional world generated on a computer screen [19]. In this case, the user is not totally immersed in the virtual world and the interaction takes place through mouse, keyboard, or joystick.

Devices such as fulldomes and embodied mixed reality learning environments (EMRELE) are able to increase the level of immersion thanks to the intensification of sensory stimuli coming from the virtual world or the enhancement of the embodiment experience in virtual interaction. By ensuring complete perceptual immersion and greater emotional transport, the user is able to interact via their body with objects in the virtual environment.

Finally, immersive virtual reality (IVR), such as head-mounted displays (HMD) and cave automatic virtual environments (CAVE), which are able to generate environments that completely surround the user and are experienced as real environments. Unlike other devices, these systems have some features that can totally absorb the user in the virtual experience such as: first-person navigation, dynamism of the scene, and stereoscopic vision.

Usually, DVRs are used in order to stimulate the interest and attention of students towards the subject studied; fulldomes and EMRELES are mainly used in collaborative learning contexts; IVRs are mainly used in the field of e-Health and education.

Thus, increasing the level of immersion increases the level of user engagement.

The adoption of VR and AR technologies in disaster medicine training can provide several benefits. Firstly, it can create a safe and controlled environment for the students, allowing them to learn and practice their skills without putting real patients at risk. Secondly, it can enable the students to experience a wide range of disaster scenarios that they may not encounter in their conventional training settings [20]. This exposure can help them develop a broad range of skills and competencies, in order to learn how to manage various disasters. Thirdly, it can enable the students to repeat scenarios until they have mastered the skills required to manage them effectively [20]. Additionally, it can enable students to experience a wide range of disaster scenarios, which can help them develop a broad range of skills and competencies [21].

However, VR and AR also have some disadvantages. The cost of equipment and software development can be a barrier to widespread implementation. Additionally, the learning curve associated with using VR and AR technologies may require additional training for educators and students. Technical issues such as system glitches or compatibility problems can also occur. Nonetheless, the benefits of using VR and AR in disaster preparedness training outweigh these challenges, making them valuable tools for enhancing the skills and readiness of healthcare professionals and students [22].

Given the breadth of today’s technological offerings, numerous types of devices have been applied in the field of training for healthcare personnel. However, to the best of our knowledge, an overview of the tools used in MCI training is lacking.

In order to systematically map the literature available on this topic, identifying the key concepts, sources of evidence, and gaps in the research, the aim of this work was to identify and analyze studies reporting VR or AR simulations regarding training for MCI among nursing and paramedic students and to investigate the adopted tools.

## 2. Materials and Methods

The scoping review allows researchers to identify the types of available evidence in a given field, clarify key concepts and definitions in the literature, examine how research is conducted on a certain topic or field, and identify key characteristics or factors related to a concept [23]. Thus, we conducted a scoping review, according to the PRISMA extension for scoping reviews (PRISMA-ScR) [24], and following the methodology of Arksey and O’Malley [25], along with recommendations by Levac and colleagues [26]. The review involved five steps: (1) Formulating the research question, (2) identifying pertinent studies, (3) selecting relevant studies, (4) extracting and organizing data, and (5) reviewing and summarizing the findings.

### 2.1. Search Strategy

The PCC method (P = population, C = concept, C = context) was used to define the research question: P = nursing or paramedic students, C = virtual reality or augmented reality simulation, and C = education and training about disaster medicine. The chosen population was extended to include paramedical staff because of the possible overlap of skills between them and nursing staff in different countries. Five databases (CINAHL-Cumulative Index to Nursing and Allied Health Literature, Cochrane Library, Pubmed, Embase, Ovid Medline) were searched for papers published with no time restrictions. The adopted search string was: (“Virtual Reality” OR “Virtual Reality Exposure Therap*” OR “Augmented Reality”) AND (“Mass Casualty Incident*” OR “Disaster Medicine”). The reference lists of all included studies were hand-searched for additional relevant reports or key terms. Targeted Internet searching using Google Scholar was also examined for additional studies of interest.

### 2.2. Eligibility Criteria

In this scoping review, papers that met the inclusion criteria were primary quantitative design studies published in peer-reviewed journals in either English or Italian. Studies focused on nursing or paramedic students, either individually or in combination, as long as their data could be extracted from the overall data, were included. The papers also should be focused on the educational or training curricula. No restrictions were adopted on the publication years of the included studies. On the other hand, papers that were excluded from the review were those that focused on students other than nursing or paramedic education, abstract-only papers, case reports, opinion papers, and books/theses.

### 2.3. Studies Selection

Titles and abstracts of the retrieved records were screened for eligibility by two reviewers (C.E.M., Y.L.) independently, to identify relevant studies on VR and simulation in disaster preparedness and triage training. Then full texts of the remaining articles were retrieved and independently assessed for inclusion based on eligibility criteria by the same two reviewers, and included for data extraction if consensus of the authors was reached (C.E.M., Y.L.). In case of disagreement, a third author (S.B.) would serve as tie-breaker. The process favored a comprehensive and unbiased selection of studies for the review.

### 2.4. Data Extraction

Characteristics of each study were extracted and synthetized, including: authors, year, country, aim(s), study design, sample, MCI setting, interventions, outcome(s), and measurements. Any disagreement between the authors in the data extraction was resolved by a consensus discussion to make the final decision [27]. The majority of included studies was heterogeneous in samples and measurement tools. Therefore, a convergent and sequential synthesis design was adopted [28,29] and thematic analysis was utilized to manage the extracted data [30]. The authors engaged in an iterative process of independent and repeated readings of the included studies to familiarize themselves with the data and extract meaningful information. Through an inductive analysis, the authors independently and critically examined the data, comparing similarities and differences, and then synthesizing the findings [29].

## 3. Results

**Figure 1:**
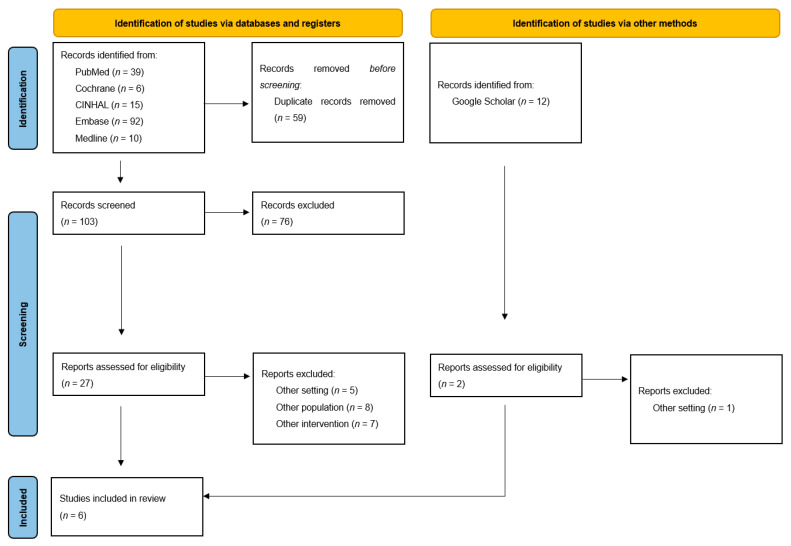
PRISMA 2020 flow diagram for new systematic reviews which included searches of databases, registers, and other sources.

The study selection process is presented in the PRISMA-ScR flow-chart (Figure 1), following the PRISMA-ScR checklist [24] (Supplementary Materials Figure S1), showing the systematic and methodological steps of this scoping review. A total of 162 potentially relevant records were identified from five databases. After the removal of duplicates (59 out of 162), 103 records were screened for eligibility based on their titles and abstracts. Following this initial screening, 25 papers were selected for further evaluation and read in detail by the authors. In addition, the authors identified 12 papers through Google Scholar and the reference lists of the included studies, two of which were also read in detail. Ultimately, six papers met the inclusion criteria and were included in this scoping review.

### 3.1. Study Characteristics

The characteristics of the included studies are presented in Table 1.

The six papers identified, published between 2016 and 2022, utilized quantitative study designs. Specifically, the designs included a quasi-experimental [31,32,33,36] and randomized controlled trial [34]. Two studies were conducted in the United States [32,33], one in Spain [31], one in Australia [36], and two in China [34,35].

### 3.2. Findings of the Qualitative Synthesis

#### 3.2.1. Methodological Aspects

All the studies explored the potential of simulation technology in disaster preparedness and triage training for students, demonstrating their effectiveness in enhancing learning outcomes, knowledge retention, and overall engagement compared to traditional methods. In all of the included studies, the intervention was performed involving VR, however, none of these adopted AR technology. The study by Ferrandini Price et al. [31] assessed the efficiency of the simple triage and rapid treatment (START) protocol during the implementation of an MCI simulation scenario and compared two different types of triage methods: VR and clinical simulation (CS). The study recruited 35 voluntary participants, who were health professionals and students attending the Official Emergency and Special Care Nursing Master’s Degree program at the Catholic University of Murcia. Smith et al. [32] conducted a quasi-experimental study to evaluate the effectiveness of two levels of immersive virtual reality simulation (VRS) in disaster education. The study involved a total of 172 out of 197 senior baccalaureate degree nursing students that completed the study. Smith et al. [33] also conducted another similar research exploring the effects of VRS on learning outcomes and retention. The quasi-experimental study enrolled 108 senior baccalaureate students. Shujuan et al. [34] conducted a randomized, controlled, single-blinded trial to evaluate the use VR disaster preparedness scenarios for nursing students. The study included 101 second-year (s-year) nursing students from a tertiary program in Sichuan. Mills et al. [36] conducted a study comparing the efficacy of simulation training between VR and live-scenario triage. The trial enrolled 29 s-year students attending a Bachelor of Paramedicine Science qualification. In the study by Hu et al. [35], the effectiveness of a VR mobile game-based application (VR-MGBA) was compared to a traditional lecture for teaching disaster evacuation management education. The study involved 158 nursing students.

#### 3.2.2. Type of Training

The included studies examined various types of training methods focused on disaster preparedness and triage training. This investigation underscored the importance of exploring different training approaches, especially those involving VR and simulation technology, in enhancing disaster preparedness and triage skills. The results collectively demonstrated the potential benefits of using VR-based methods, which led to improved learning outcomes, better retention of knowledge, and increased engagement, compared to traditional instructional techniques. Ferrandini Price et al. [31] conducted the VR simulation using a VR device equipped with a head-mounted display (HMD) to fully immerse participants in the VR content, while the CS simulation used actors to simulate real-life scenarios during the training. In Smith et al. [32], participants were randomly assigned to one of the three groups: (i) immersive HDM VRS (*n* = 59), (ii) keyboard and mouse VRS (*n* = 58), or (iii) a control group consisting of written instruction (*n* = 55). Before their respective interventions, all three groups completed a 30 min web module explaining decontamination skills. Similarly, Smith et al. [33] randomized participants into two groups: the treatment group, which received VRS (*n* = 57), and the control group, which received written instruction (*n* = 51). Both groups watched a 25 min web module explaining decontamination skills prior to beginning the study. Shujuan et al. [34] randomly allocated participants into two groups: a VR group (*n* = 49) and a control group (*n* = 52). In the study by Mills et al. [36], participants were randomized into either VR or live simulation. Finally, in Hu et al. [35], 78 students were assigned to the game group and 80 to the lecture group.

#### 3.2.3. Types of Measurements

All the studies employed various types of instruments for data collection to assess the effectiveness of different training methods in disaster preparedness and triage. Studies employed a wide range of measurement instruments, such as VR devices, HDMs, written instruction, performance rubrics, cognitive tests, questionnaires, simulation evaluations, physiological measurements, and satisfaction surveys, providing comprehensive and valuable data to understand the effectiveness of VR and simulation-based disaster preparedness and triage training methods. Ferrandini Price et al. [31] used a VR device with an HDM and CS with actors for their triage training comparison. Data collection involved simulation exercises and performance of basic triage on 20 victims using the START protocol evaluations to measure the participants’ preparedness and performance in life-saving maneuvers such as opening the airway and applying hemorrhage compression during an MCI. Smith et al. [32] utilized multiple instruments to evaluate disaster education methods. They employed immersive VR simulations with HDM, keyboard and mouse VRS, and traditional written instruction. The data collection included a demographic questionnaire to understand participants’ characteristics, performance rubrics to assess their performance, cognitive learning tests to gauge knowledge acquisition, and focus groups to gather qualitative insights from the participants. Similarly, Smith et al. [33] used VRS and written instruction for disaster education and employed performance rubrics and cognitive learning tests for data collection to evaluate the participants’ learning outcomes and knowledge retention. Shujuan et al. [34] used VR with HDMs for disaster preparedness scenarios and compared it with a traditional disaster nursing course. Data collection involved questionnaires to assess the participants’ experiences and knowledge, as well as simulation performance evaluations to gauge their disaster preparedness skills. Mills et al. [36] conducted a study comparing VR and live-scenario triage simulation training. They collected data through various instruments, including heart rate measurements to assess physiological responses during the training, NASA-TLX (Task Load Index) to gauge mental workload, clinical decision-making evaluations to assess participants’ decision-making skills, satisfaction questionnaires to gather feedback on their experiences, and a cost analysis to evaluate the cost-effectiveness of the training methods. Hu et al. [35] compared a VR-MGBA with traditional lectures for disaster evacuation management education. Data collection included multiple-choice questions and Likert-scale type questions to evaluate participants’ knowledge and opinions through an instructional mode opinion survey.

## 4. Discussion

The aim of this scoping review was to provide a comprehensive overview of the adopted tools for VR or AR simulations regarding training for MCI, among nursing and paramedics students.

In fact, numerous devices have been used in the training of healthcare personnel, given the advances in technological development in this field. However, this aspect can be dispersive for professionals wishing to adopt such training. This paper provides an overview of VR and AR tools adopted in literature.

VR and AR represent an emerging approach, which utilizes technology to enhance the training of nursing and paramedic students in many fields including MCI.

All the included studies in this review adopted VR technology, and found that it had an overall positive impact and the potential to address the need for competency development in disaster medicine education, as suggested by Shunjuan et al. [34], and may be more effective than other training methods, as found by Hu et al. [35]. While some studies [31,32,33,36] have not demonstrated the superiority of VR over traditional methods in training nursing and paramedic students, VR has been shown to be more efficient [31], cost-effective [32,33,34,36], easy to access [32,33], and safe [32,33] for disaster medicine training.

This result is consistent with recent systematic reviews on the topic [37,38].

As these studies adopt different MCIs’ settings, VR has been shown to be representative of many possible scenarios, facilitating more all-encompassing training [39]. AR technology still appears to be underutilized, although there is evidence to show that they are accepted and viewed as beneficial by students [40].

As reported above, higher levels of immersion are associated with higher levels of self-efficacy [14].

This is an important aspect, as self-efficacy reinforcement was identified as the most crucial component of resistance against experiencing high amounts of stress [41].

The included studies did not fully address this aspect, except for Mills and colleagues [36], but they represent a good starting point for further investigation of this parameter in future.

Nevertheless, there is evidence that there is a sizable gap between nurses’ opinions of their own levels of competencies and their actual levels of competencies [42].

For this reason, VR training has been shown to be an effective way to highlight awareness of this discrepancy [43].

Despite the potential benefits of this technology in disaster medicine training, there are some challenges and limitations that need to be addressed. Firstly, there is a lack of standardization in VR technology, which can affect the quality and consistency of training across different institutions [21]. In addition, all studies included different methods to assess the efficacy of the adopted intervention, limiting the possibility to compare the results.

Another limitation is the cost of implementing VR or AR technology and the need for technical support and expertise to develop and maintain VR simulations. This can be a challenge for institutions that do not have the resources to support such technology [21]. However, as shown by our results, there are different types of adoptable tool, resulting in different price ranges.

For this reason, further study should assess the cost-effectiveness of different types of interventions, while considering the level of immersion achieved.

### Strengths and Limitations

To the best of our knowledge, the strength of this study lies in it being the first to provide an overview of the tools and settings of VR and AR for nursing training in MCI. This study has some limitations. First, this article does not perform a critical assessment of the literature included. However, as a scoping review, the aim of this study was not to synthetize evidence, but to pool together elements and core concepts from various bodies of knowledge. Conducting a critical assessment of the literature included would strengthen the validity and reliability of the results. Additionally, exploring the quality and biases of the individual studies could provide a clearer picture of the strengths and weaknesses of the evidence gathered. The literature review was performed until February 2023, exposing this work to a publication bias. Finally, the included studies were highly inhomogeneous. As the field of VR and AR in nursing training for MCI is rapidly evolving, future research could explore emerging technologies and novel applications. Investigating the long-term impact of VR and AR training on nursing students’ performance and patient outcomes could be another avenue for future exploration. Despite the identified limitations, this study provides valuable insights into the current landscape of VR and AR applications in students training for MCI scenarios, thus promoting the dissemination and implementation of the retrieved experiences. By addressing the suggested improvements and exploring future research opportunities, this field can continue to advance, leading to more effective and innovative training approaches for students in disaster response and triage situations.

## 5. Conclusions

To date, it is still challenging to state which method of delivering disaster education or preparedness training and their effectiveness within specific contexts and populations is the most effective, as each study focuses on different strategies. However, VR and AR methods have been shown to potentially improve initial skills development and engagement. Furthermore, they may be more cost-effective and efficient ways to deliver training compared to traditional methods such as lectures or written instruction, and the choice of methods and tools will depend on the specific needs and goals of the training program, the target population, and the available resources. There is an increasing need to enhance the use of VR and AR in the educational paths of nursing students. Further research are needed to determine the effectiveness of different VR methods or their long-term retention of knowledge and behavioral effects.

## Figures and Tables

**Table 1:** Included studies.

Authors and Year	Country	Aim(s)	Study Design	Sample	MCI Setting	Interventions	Outcome(s)	Measurements
Ferrandini Price et al. (2018) [31]	Spain	To determine the efficiency in the execution of the START (simple triage and rapid treatment) triage, comparing VR to CS in an MCI. To compare the stress caused by the two different situations.	Quasi-experimental study	Sixty-seven students attending the Official Emergency and Special Care Nursing Master’s Degree from the Catholic University of Murcia.	Performing basic triage in all the victims using the START system, including life-saving maneuvers: airways opening and hemorrhage compression.	Simulation with actors; Immersion with virtual reality.	Stress and activation; Efficacy in the performing of the START triage.	Saliva collection through a system of passive diffusion, in a tube, with an extraction time of 1 min; Analysis of the answers and teacher evaluation.
Smith et al. (2018) [32]	United States	To assess two levels of immersive VR simulation to teach the skill of decontamination.	Quasi-experimental study	Of a total of 197 senior baccalaureate nursing students from four Midwest campuses, 172 completed all three testing periods in the study.	Decontamination skills.	Web module + immersive HMD VR simulation; Web module + keyboard/mouse VR simulation; Web module + written instruction.	To assess levels of performance and time; To assess level of knowledge; To assess participant satisfaction and experiences.	Pretest: demographic questionnaire and baseline cognitive test; Post-test (after decontamination training): cognitive test and demonstration checklist; Post-test II (after 6 months from training): cognitive learning assessment and demonstration checklist. Focus group about participant’s satisfaction and experience with the two different VR simulation formats.
Smith et al. (2016) [33]	United States	To examine the longitudinal effects of VR simulation on learning outcomes and retention.	Quasi-experimental study	108 students (57 were in the treatment group and 51 in the control group).	Decontamination skills.	Web training + written instruction, Web training + VR simulation.	To assess accuracy and time to complete the psychomotor performance; To measure cognitive learning.	Psychomotor performance (17-item performance rubric), Cognitive learning (20 multiple-choice questions).
Shujuan et al. (2022) [34]	China	To assess the impact of VR scenarios on disaster preparedness among nursing students.	A two-arm randomized controlled trial	101 nursing students (49 in the VR group and 52 in the control group) attending the second year of a tertiary program in Sichuan, China.	Twelve highly interactive disaster scenarios: Earthquake Fire Triage Wound Dressing Fixation Hemostasis Debridement Cardiopulmonary resuscitation Tracheal intubation Transportation Decontamination Supportive psychological care	Intervention group: usual disaster preparedness nursing course and VR training scenarios which included 12 highly interactive disaster nursing scenarios. Each scenarios included two disaster scenes (earthquake and fire), triage, wound dressing, fixation, hemostasis, debridement, cardiopulmonary resuscitation, tracheal intubation, transportation, decontamination, and supportive psychological care, and also included an instructing model, training model, and testing model. Control group: usual disaster nursing course which included 24 lectures and 4 skills laboratory manikin simulation sessions.	To assess disaster preparedness in triage, communication, isolation, psychological support and decontamination, report, and access to important resources; To assess level of self-confidence; To assess the performance of simulated disaster incident.	The following data collection were assessed at baseline and at the end of the study. DPQ (30 items using a 5-point Likert scale); Confidence (self-developed assessment cards using a scale ranged from 0 to 9); Performance (assessed by 5 examinators).
Hu et al. (2022) [35]	China	To explore the effectiveness of a virtual reality mobile game-based application for teaching disaster evacuation management education to nursing students.	Quasi experimental study	158 nursing students (78 in the game group and 80 in the lecture group).	Three disaster situations: fire scenario, earthquake scenario, first aid scenario.	Game class: pre-test, basic knowledge and skills, mobile game learning (1 h basic knowledge lecture, 4 h for VR-MGBA use, 1 h learning summary), post-test, final-test, and questionnaires; Lecture class: pre-test, basic knowledge and skills, traditional lecture (6 h lecture class), post-test, final-test, and questionnaires.	To assess essential disaster evacuation management educational knowledge and decision-making abilities; To assess students’ opinion.	Pre-test before the educational intervention (20 multiple-choice questions); Post-test following the intervention (20 multiple-choice questions); Final-test at the end of the term (20 multiple-choice questions); Opinion survey (5-point Likert-scale-type questions).
Mills et al. (2019) [36]	Australia	To compare the simulation efficacy of a bespoke virtual-reality MCI simulation with an equivalent live simulation scenario designed for undergraduate paramedicine students.	Quasi-experimental study	29 students of paramedical science.	Car chase and shoot.	VR simulation; Live simulation with actors.	To assess: immersion, clinical decision-making, learning satisfaction, and cost.	Immersion (recording heart rate at 5 s intervals and through the 20-point scales NASA-TLX across mental, physical, temporal, performance, effort, and frustration); Clinical decision-making (through educator evaluation); Satisfaction (using the 20-item SDS assessing perception of information, support, problem solving, feedback, and fidelity using a 5-point Likert scale); Focus group (two focus groups with 8 participants of both VR and live simulation groups); Cost analysis (using the Maloney and Haines methods).

Legend. CS: clinical simulation; DPQ: Disaster Preparedness Questionnaire; HMD: head-mounted display; MCI: mass casualty incident; MGBA: mobile game-based applications; NASA-TLX: National Aeronautics and Space Administration Task Load Index; SDS: simulation design scale; VR: virtual reality.

## Supplementary Materials

The following supporting information can be downloaded at: https://www.mdpi.com/article/10.3390/bs13070616/s1, Figure S1: PRISMA-ScR checklist.
